# Physical Fitness with Regular Lifestyle Is Positively Related to Academic Performance among Chinese Medical and Dental Students

**DOI:** 10.1155/2020/5602395

**Published:** 2020-01-16

**Authors:** Yujiao Hou, Guang Mei, Yutong Liu, Weisheng Xu

**Affiliations:** College of Electronics and Information Engineering, Tongji University, Shanghai, China

## Abstract

**Objective:**

The purpose of this study was to examine the relationship between physical fitness, lifestyle, and academic performance of Chinese college students and investigate the differences among medical and dental students on their lifestyle.

**Methods:**

This study was conducted with 316 students enrolled from 2012 to 2014 at Tongji University. Scores from the college physical test were used to represent the students' physical fitness condition. Lifestyle was measured by some variables extracted from the students' behavior data provided by the university's information center. Academic performance was measured by the average score of basic courses and the average score of professional courses. Demographic information, including age, gender, nation, and family background, was also obtained. Separate multiple linear regression analysis was performed for modeling academic performance and physical fitness with a *p* value threshold of 0.05.

**Results:**

A total of 212 (45.97% females) medical students and 104 (58.65% females) dental students participated in this study. Physical fitness score (medical: *r* = 0.34, *p* value threshold of 0.05. *r* = 0.34, *p* value threshold of 0.05. *r* = 0.34, *p* value threshold of 0.05. *r* = 0.34, *p* value threshold of 0.05. *r* = 0.34, *p* value threshold of 0.05. *r* = 0.34, *p* value threshold of 0.05. *r* = 0.34, *p* value threshold of 0.05. *r* = 0.34, *p* value threshold of 0.05. *r* = 0.34, *p* value threshold of 0.05. *r* = 0.34, *p* value threshold of 0.05. *r* = 0.34, *p* value threshold of 0.05. *r* = 0.34, *p* value threshold of 0.05. *r* = 0.34, *p* value threshold of 0.05. *r* = 0.34, *p* value threshold of 0.05.

**Conclusion:**

Physical fitness, library usage, and the regularity of lifestyle are significant contributors to academic performance among Chinese medical and dental students. Moreover, medical students are shown to have less rest time compared to dental students.

## 1. Introduction

Physical fitness was found to have a close relationship with young people's cognitive ability, self-control, executive function, memory, and so on [1–7]. For college students, these abilities are critical to academic performance. There are plenty of researches who have explored the direct correlation between physical fitness and academic performance among children and adolescents. Pindus et al. found that aerobic fitness was positively associated with the childhood ability to manage perceptual interference and spelling [8]. For preadolescent children, positive effects were found for physical activity on academic performance (*g* = 0.26; 95% CI = 0.02 and 0.49; 3 studies) [9]. Likewise, high fitness performance in adolescence was also associated with higher subsequent academic achievement [10]. However, there are few studies that focus on the relationship between physical fitness and college students' academic performance. A health-related physical fitness test is a valid way to measure the level of human physical fitness. Globally, there are various commonly used multistage fitness test batteries in student's physical fitness evaluation, such as FitnessGram [11], Eurofit [12], and Alpha-fit [13]. The FitnessGram is a valid and reliable indicator of health-related fitness especially aerobic capacity of school students [14]. However, in Chinese universities, the annual physical fitness test is required, and in which, five components of health-related physical fitness were measured: (1) morphological components: height, weight, and body mass index (BMI); (2) musculoskeletal components: standing long jump test and crunch/pull up; (3) motor component: speed test (50 meter run); (4) flexibility component (hamstring and lumbar extensibility, sit-and-reach test); (5) cardiorespiratory components: vital capacity and 800 meter/1000 meter run test to estimate maximal oxygen consumption [15]. The physical fitness tests are performed twice a year, at the 16th week of each semester, and it gives the opportunity to examine the relationship between students' physical fitness and academic performance.

Medical education is one of the most challenging, demanding, and stressful fields of study since students are expected to acquire diverse academic and clinical competencies and interpersonal skills. Indeed, medical students have heavier academic pressure and less leisure time compared to students in other fields. The effect of lifestyle on the academic performance of college students has been studied for years. Eliasson et al. [16] examined the importance of sleep time for academic performance among college students and found that students with the highest performance had significantly earlier bedtimes (*p*=0.05) and wake times (*p*=0.008). Faught et al. [17] found that healthier diet including more protein-rich foods, vegetables, and fruits might help on higher academic achievement among youth. Wang et al. [18] investigated the importance of student employment in predicting academic performance. However, these existing researches mostly relied on questionnaires in collecting students' lifestyle information. With the development of technology, students' daily behavior data are more and more collected by electronic devices. In China, especially, university students have their smartcards for purchasing in campus's canteens, stores, and for access to libraries and dormitories. In Tongji University, the records of students swiping smartcards were stored in the Information Center, and based on these records, we can analyze students' daily behavior by simulating the situation of swiping cards and concluding the rules of behavior. It is more precise and insightful than investigating students' behavioral habits by questionnaires.

Several studies have been conducted comparing physical fitness, lifestyle, sociodemographic factors, and academic performance of college students, but due to the sociocultural discrepancies, findings of other countries cannot be extrapolated to the Chinese population. Furthermore, no study has been previously conducted in China comparing academic performance with physical fitness and lifestyle. This study was planned to find the association between academic performance with different demographic factors, physical fitness, and lifestyle amongst Chinese medical and dental students.

## 2. Materials and Methods

This study was approved by the Big Data and Network Security Research Center of Tongji University. The usage of students' courses grades and smartcard swiping data was approved by Tongji University Information Center. Sensitive personal information extraction and usage guidelines were strictly followed during the study period.

### 2.1. Participants

The participants were 212 medical students and 104 dental students enrolled from 2012 to 2014. The number of medical students enrolled in 2012, 2013, and 2014 was 55, 77, and 80, respectively. The number of dental students enrolled in 2012, 2013, and 2014 was 33, 36, and 35, respectively. All the participants completed the questionnaire about sociodemographic factors and fitness tests. Besides, they were asked to provide their student ID for us to link their course grades. We explained the purpose of the study to the participants and confirmed that participation was voluntary with no penalty incurred in case of no participation. All participants provided informed consent to this study.

### 2.2. Data

There were four aspects of the data resource. The grades of final examinations were provided by the educational administration system database. The behavior data of students were extracted from smartcard swiping records. Physical fitness was measured by the scores of fitness tests. The data of sociodemographic factors were collected by a questionnaire.

In China, medical and dental undergraduates usually pursue four years of study with some clinical practice in the fourth year and start clinical internship officially in the final year. Hence, the assessment of their academic performance on courses should focus on the first three years. In this study, we analyzed the performance of basic courses and professional courses separately. As for basic courses, we chose 8 subjects which were required courses for both medical and dental students, including Medical Physics, Medical Mathematics, Organic Chemistry (Medical), Basic Chemistry, Topographic Anatomy, Physiology, Pharmacology, and Molecular Genetics. As for professional courses, we chose 29 subjects for medical students, including Introduction to Psychology and Behavior Science, Introduction to Clinical Medicine, Neurobiology, Pathogen Biology and Infectious Diseases, Immunology and Immune System Disease, Pathology and Pathophysiology, Medical Ethics, Epidemiology and Statistics, Traditional Chinese Medicine, Preventive Medicine, General Surgery, Comprehensive Diagnostics, Otolaryngology Science, Dermatology and Venereology, Ophthalmology, Digest Science, Cardiovasology, Obstetrics and Gynecology, Breathing Epidemiology, Emergency Medicine, General Medicine, Haematology, Neurology, Kinesiology, Integrated Diagnostics, Psychiatry, Urinary Epidemiology, Pediatrics, and Endocrine Epidemiology; 24 subjects for dental students, including Introduction to Oral Medicine, Medical Microbiology, Medical Immunology, The Application of Oral and Maxillofacial Anatomy, Oral Tissue Embryology, Oral Pathology, Internal Medicine (1), Surgery (1), Diagnostics, Dental Anatomy, Oral Physiology, Oral and Maxillofacial Medical Diagnostics, Dental Materials, Oral and Maxillofacial Surgery (1), Cariology and Endodontics, Dental Prosthetics (1), Oral Preventive Medicine, Orthodontics, Children's Oral Medicine, Oral and Maxillofacial Surgery (2), Periodontal Disease and Oral Mucosal Epidemiology, Dental Prosthetics (2), Surgery (2), and Internal Medicine (2). All course grades ranged from 0 to 100 (60 was “pass”).

The behavioral habits were extracted from the smartcard swiping records. The smartcard is the only way to purchase in canteens, visit the library, borrow books, and get in/out of the dormitories. Based on these records of the first three years after students' enrollment, we extracted three kinds of variables, including study/reading habits, rest habits, and dietary habits. The study/reading habits consisted of the number of times of visiting the library, the number of days of visiting the library, the number of borrowing books, the times of returning books overdue, the ratio of returning books overdue, and the average overdue days. The rest habits consisted of the times of wake up early (before 6:00 am), the times of staying out late (after 11:00 pm), and the times of staying out all night. The dietary habits consisted of the times of eating breakfast (during 5:00 am and 9:00 am) and the number of days in which more than two meals happened.

Physical fitness was measured by the scores of fitness tests of every semester which composed 5 parts, including BMI (accounts for 15 points), vital capacity (accounts for 15 points), standing long jump (accounts for 10 points), sit and reach (accounts for 10 points), pull up (for male)/crunches (for female) (accounts for 10 points), 50 meter run (accounts for 20 points), and 1000 meter run (for male)/800 meter run (for female) (accounts for 20 points). BMI is an indirect measure of body composition, and it is calculated as body weight in kilograms divided by the square of height in meters [19]. Commonly accepted BMI ranges are underweight: under 18.5 kg/m^2^, normal weight: 18.5 to 25, overweight: 25 to 30, and obese: over 30 [20]. However, due to the ethnic differences, for Asian people, BMI is a little bit lower. According to the State Students Health Standards of China, the BMI ranges for male are underweight: under 17.8 kg/m^2^, normal weight: 17.9 to 23.9, overweight: 24 to 27.9, and obese: over 28; for female are underweight: under 17.1 kg/m^2^, normal weight: 17.2 to 23.9, overweight: 24 to 27.9, and obese: over 28. Students whose BMI indicates normal get 10 points, while underweight and overweight get 8 points and obese get 6 points [21]. Except for BMI, the grading standards of the fitness test are shown in Table 1. Students' scores were reported as the total values which were summed by the weighted score of each subject.

The basic information besides family background was collected by a designed questionnaire which was distributed after the students were informed about the nature of the research and assured that their responses would be confidential. The content of the questionnaire included questions about age, gender, ethnicity, family income, parents' educational levels, and whether there were medical professionals among their family members. Participation was voluntary, and all the participants completed it.

### 2.3. Data Analysis

Continuous variables were represented by means and standard deviations (i.e., age as “X3,” family income as “X4,” the number of times of visiting the library in the first 3 years as “X7,” the number of times of borrowing books from the library as “X8,” the number of days of visiting the library as “X9,” the number of times of returning books overdue as “X10,” the ratio of returning books overdue as “X11,” the mean duration of returning books overdue as “X12,” the number of times of leaving dormitory before 6:30 am as “X13,” the number of times of returning to dormitory after 11:00 pm as “X14,” the number of times of leaving or returning dormitory between 1:00 am and 5:00 am as “X15,” the number of days of having meals more than twice in the canteen as “X16,” the number of days of having breakfast in the canteen as “X17,” and the scores of fitness test as “Z1”), while categorical variables are presented as absolute and relative frequencies (i.e., gender as “X1,” nation as “X2,” parents' educational level as “X5,” and number of medical practitioners among parents as “X6”). The mean grades of basic courses (Y1) and the mean grades of professional courses (Y2) are used to represent students' academic performance. Spearman's correlations were conducted as an exploratory analysis to examine bivariate associations between the variables. Four multiple linear regression models were performed to examine the relationships between physical fitness as well as life habits to the academic performance (Y1, Y2) of dental and medical students. To further investigate the effective factors on physical fitness (Z1), two additional multiple linear regression models were developed. Any *p* values equal to or less than 0.05 were considered statistically significant. Python 3.7 was used to perform all these statistical analyses.

## 3. Results

Two-hundred eleven medical students and one-hundred four dental students completed the survey and fitness tests. Descriptive statistics are shown in Table 2. The mean age of medical students was similar to the mean age of dental students. The ratio of female students in dental students was higher than the ratio in medical students. However, it was common that the academic performance of female students was much better than male students, both for medical and dental students.

### 3.1. Differences between Medical and Dental Students

As for basic information, the general family income of medical students was much lower than dental students. Correspondingly, the parents' educational levels of dental students were higher than medical students.

**Figure 1:**
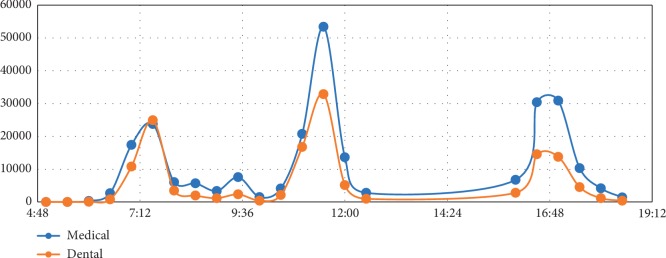
Distribution of eating in the canteens.

To analyze the life regularity of medical and dental students, we divided a day into 48 periods and counted the frequency of activities in each period. The time distribution of eating in the canteens of the two groups is shown in Figure 1. There were common peaks for both medical and dental students which are from 7:30 to 8:00, from 11:30 to 12:00, and from 16:30 to 17:30, corresponding to breakfast, lunch, and dinner, respectively. Besides, it showed that dental students' meal time was more concentrated, and they had much more enthusiasm for breakfast, compared to medical students.

**Figure 2:**
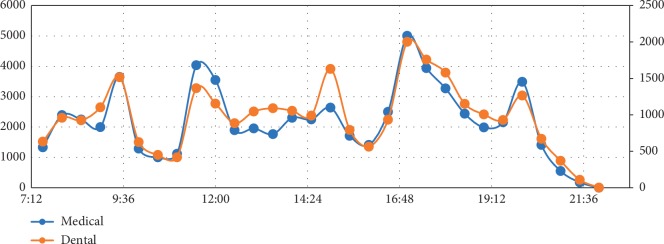
The distribution of visiting the library.

The time distribution of visiting the library is shown in Figure 2. The trends of the two groups were similar, and there were 6 common peaks on visiting the library for both medical and dental students which were from 8:30 to 9:00, from 9:30 to 10:00 (the second class of a day dismissed at 9:35), from 11:30 to 12:00 (the fourth class of a day dismissed at 11:35), from 15:00 to 15:30 (the sixth class of a day dismissed at 15:05), from 17:00 to 17:30 (the eighth class of a day dismissed at 17:05), and from 20:00 to 20:30 (the tenth class of a day dismissed at 19:45). In addition, the mean number of times of visiting the library of medical students (306.0 times) was much bigger than dental students (278.8 times). But due to the fact that students need to swipe their smartcard only when they were entering the library and they can leave freely without swiping cards, we cannot calculate the duration of every visit. Hence, we could not infer that medical students spent more time than dental students in the library. It might show that medical students' courses and experiments were more fragmentary which made students have to get in and out of the library many times in a day.

**Figure 3:**
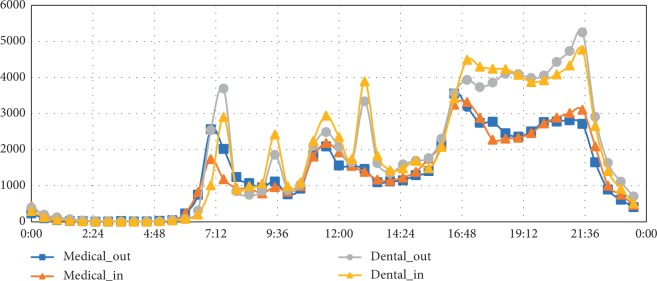
The distribution of getting in/out of the dormitories.

The time distribution of getting in and out of the dormitories is shown in Figure 3. The number of medical students was more than twice the number of dental students. But dental students' frequency of getting in and out of dormitories was almost always higher than the frequency of medical students, especially at mealtime, the time after the second class dismissed, the lunch break, and the whole evening. Besides, it turned out that medical students got out earlier than dental students (the first peak of medical students was earlier to dental students) and had fewer night activities than dental students. The results might suggest that medical students cannot get enough rest time compared to dental students.

### 3.2. Lifestyle, Physical Fitness, and Academic Performance

Spearman's correlations among variables are shown in Table 3. To further examine the relationship between lifestyle, physical fitness, and academic performance, six multiple linear regression models were developed. The grades of basic courses, the grades of professional courses, and the scores of fitness tests were used as the dependent variables, separately. The coefficients and performance of the models are shown in Table 4. The combination of these variables can explain 23.7% of the variance in basic course grades for medical students, 39.6% of the variance in professional course grades for medical students, 58.0% of the variance in basic course grades for dental students, and 43% of the variance in professional course grades for dental students. And the variables of basic information and lifestyles can explain 30.3% of the variance in physical fitness for medical students and 34.6% of the variance physical fitness for dental students.

Next, six multiple linear regression models were developed to further examine the relationship between lifestyle, physical fitness, and academic performance. The grades of basic courses, the grades of professional courses, and the scores of fitness tests were used as the dependent variables, separately. The coefficients and performance of the models are shown in Table 4. The combination of these variables can explain 23.7% of the variance in basic course grades for medical students, *R*^2^ = 0.24, RMSE = 72.36, *p* < 0.001, 39.6% of the variance in professional course grades for medical students, *R*^2^ = 0.40, RMSE = 36.97, *p* < 0.001, 58.0% of the variance in basic course grades for dental students, *R*^2^ = 0.58, RMSE = 51.13, *p* < 0.001, and 43% of the variance in professional course grades for dental students, *R*^2^ = 0.43, RMSE = 59.95, *p* < 0.001. The variables of basic information and lifestyles can explain 30.3% of the variance in physical fitness for medical students, *R*^2^ = 0.30, RMSE = 50.81, *p* < 0.001, and 34.6% of the variance physical fitness for dental students, *R*^2^ = 0.35, RMSE = 42.16, *p* < 0.001.

## 4. Discussion

This study aimed to evaluate the relationship between physical fitness, lifestyle, and academic performance among medical and dental students. The results showed that high fitness test scores accompanied by regular life had a positive effect on academic performance both for medical and dental students. Besides, the differences in life behavior between medical students and dental students were investigated which was few studies had examined before. The behavior data showed that medical students had less breakfast, rest time in the dormitory, but visiting the library was more frequent, compared to dental students. Based on these observations, we inferred that medical students were under heavier academic pressure than dental students.

The factors that impact academic performance were examined, and finally, three factors were found to be the most significant: the number of times of eating in the canteens, the times of visiting the library, and physical fitness. There had been several studies conducted compared to academic performance with dietary habits, but nearly all of them were among children and adolescents. Researchers found that breakfast consumption may improve cognitive function related to memory, test grades, and school attendance among European children and adolescents [22–24]. Recently, breakfast eating behavior was also found significantly relative to academic performance among Bahria University students [25]. However, in 1997, Fekete and Head found breakfast consumption has no attribute on test grades of college students [26]. In our study, breakfast eating frequency was found to be significantly associated with academic performance and physical fitness among medical and dental students. The result was consistent with the opinion of Rehman's study which was conducted in 2018.

As for library material usage, there was an obvious difference between medical students and dental students. The general frequency of visiting the library of medical students was slightly higher than dental students'; however, the general frequency of borrowing books from the library of medical students was much lower than dental students. Besides, the frequency and number of days of visiting the library were found to be significant influencing factors to dental students' academic performance, which was not found among medical students. There are a few studies suggesting that library usage had a positive effect on academic performance [27, 28]. Jan et al. conducted an interesting study about library anxiety, library use, and academic performance in Pakistan and found that students who visited the library frequently had less library anxiety and better academic performance [29]. Twenty years before, Brazier and Conroy analyzed the relationship between the number of books borrowed and examination marks in the premedical year and the fifth medical year of medical students in a medical school in Ireland, and they found that the patterns are totally different between these two years [30]. There was significant relevance between the level of student borrowing and examination marks during the premedical year, but no such association was found during the fifth medical year. In our study, the frequency of visiting library and the number of borrowing books was found to have significant relationships with students' academic performance (both medical students and dental students). By contrast, the behavior of returning books overdue was basically not found to be relative to students' academic performance.

Physical fitness was a commonly admitted key factor to people's physical and mental health, and it was found to be strongly relevant to the academic performance of children and adolescents [31–34]. Also, researchers did a few studies on the impact of physical fitness on college students. Lipošek et al. did research in the University of Maribor and found that periods of two to three hours of weekly physical activity were positively associated with academic success [35]. Qi et al. investigated the associations of physical activity and screen time with depression, anxiety, and sleep quality among Chinese college freshmen and found that physical activity can reduce the prevalence of depression and help sleep quality [36]. A cross-sectional study was conducted by Al-Drees et al. to explore the relationship between physical activity habits and GPA of medical students in Saudi Arabia [37]. And the result showed that physical activity was positively associated with GPA. In our study, we adopted 7 typical physical measurements to represent the physical fitness condition of students. It was consistent with former studies that better physical fitness can contribute to students' academic achievement, for both medical and dental students.

In addition, we found many correlations within students' behavior. First, the frequency of visiting the library and the frequency of eating in the canteens had a strong correlation. We can infer that students with a high frequency of visiting the library and eating in the canteens live a regular life with self-discipline, and these students are more likely to have good grades and a healthy body. Meanwhile, male and female dental students were rather different in terms of rest time. In detail, female students tended to have less time spending in their dormitories because the results showed that “female” had a significant effect on “get out early in the morning” (*p* < 0.001) and “return late in the evening” (*p* < 0.001). However, these differences did not exist among medical students. Instead, for medical students, those who had a higher frequency of “get out early in the morning” and “return late in the evening” also had a higher frequency of visiting the library and eating in the canteens, which was not found significant among dental students. Besides, through the comparison of the distributions of getting in/out of dormitory between medical students and dental students, we found that medical students' time of “get out of the dormitory in the morning” was generally earlier than dental students, and their time of “return back to dormitory in the evening” was also later than dental students. Considering all the differences on rest time between the two kinds of students, we inferred that due to high academic pressure, medical students all had to wake up early and get back late and even sacrifice their lunch break so that they could have enough time to handle their study, no matter male or female.

However, this study had some limitations as well. The number of dental students was not enough. And another important defect was that there were missing records of students' swiping cards when they got in/out of the dormitory. Due to the design of the door control system, once the door was opened after someone swiped the card, it took about 3 seconds to close. Hence, a student can get in/out of the dormitory without swiping the card as long as the door was not closed yet. At some crowded time, many students did not have to swipe their cards to go through the door. Indeed, we can only have rough numbers to analyze students' activity in dormitories. So as the missing records of leaving the library. Moreover, given that participants were sampled from a university setting, caution is urged in generalizing these findings to other populations.

The strengths of this study should also be acknowledged. This study used real behavior data to analyze students' lifestyle, providing reliable preliminary evidence of objective associations between lifestyle and academic performance. This is the first study that provides initial evidence of Chinese university students' physical fitness, lifestyle, and academic performance, which may have implications to further research. According to the findings of this study, the university should guide students to balance their academic, rest time, and body fitness more scientifically.

## Figures and Tables

**Table 1:** Grading standards of the fitness test.

Score	Vital capacity (ml)		Standing long jump (cm)		Sit and reach (cm)		Pull up (times)	Crunches (times)	50 meter run (s)		1000/800 meter run	
	Male	Female	Male	Female	Male	Female	Male	Female	Male	Female	Male	Female
10	5040	3400	273	207	24.9	25.8	19	56	6.7	7.5	3′17″	3′17″
9.5	4920	3350	268	201	23.1	24	18	54	6.8	7.6	3′22″	3′22″
9	4800	3300	263	195	21.3	22.2	17	52	6.9	7.7	3′27″	3′27″
8.5	4550	3150	256	188	19.5	20.6	16	49	7	8	3′34″	3′34″
8	4300	3000	248	181	17.7	19	15	46	7.1	8.3	3′42″	3′42″
7.8	4180	2900	244	178	16.3	17.7		44	7.3	8.5	3′47″	3′47″
7.6	4060	2800	240	175	14.9	16.4	14	42	7.5	8.7	3′52″	3′52″
7.4	3940	2700	236	172	13.5	15.1		40	7.7	8.9	3′57″	3′57″
7.2	3820	2600	232	169	12.1	13.8	13	38	7.9	9.1	4′02″	4′02″
7	3700	2500	228	166	10.7	12.5		36	8.1	9.3	4′07″	4′07″
6.8	3580	2400	224	163	9.3	11.2	12	34	8.3	9.5	4′12″	4′12″
6.6	3460	2300	220	160	7.9	9.9		32	8.5	9.7	4′17″	4′17″
6.4	3340	2200	216	157	6.5	8.6	11	30	8.7	9.9	4′22″	4′22″
6.2	3220	2100	212	154	5.1	7.3		28	8.9	10.1	4′27″	4′27″
6	3100	2000	208	151	3.7	6	10	26	9.1	10.3	4′32″	4′32″
5	2940	1960	203	146	2.7	5.2	9	24	9.3	10.5	4′52″	4′52″
4	2780	1920	198	141	1.7	4.4	8	22	9.5	10.7	5′12″	5′12″
3	2620	1880	193	136	0.7	3.6	7	20	9.7	10.9	5′31″	5′32″
2	2460	1840	188	131	−0.3	2.8	6	18	9.9	11.1	5′52″	5′52″
1	2300	1800	183	126	−1.3	2	5	16	10.1	11.3	6′12″	6′12″

**Table 2:** Descriptive data for all variables.

	Medical		Dental	
Age in years (X3), M (SD)	18.60	0.77	18.45	0.84
X4	4166.35	3697.00	4766.35	3254.41
X7	356.05	370.28	334.32	331.25
X8	23.10	33.02	29.19	41.63
X9	180.34	143.12	174.81	131.04
X10	4.37	6.52	4.74	8.94
X11	0.19	0.24	0.18	0.26
X12	47.38	163.39	33.14	63.60
X13	10.38	15.65	7.66	12.08
X14	16.94	26.30	45.77	50.94
X15	6.27	12.93	14.35	19.49
X16	195.61	165.51	164.63	125.10
X17	234.62	205.16	255.06	152.19
Z1	82.91	8.56	84.10	8.07
Y1	66.45	9.76	77.50	11.08
Y2	79.87	7.84	77.14	10.30
Female, *n* (%)	97 (45.97)	61 (58.65)		
Ethnicity, *n* (%)				
Han	181 (85.78)	94 (90.38)		
Minority	30 (14.22)		10 (10.64)	
Parents' educational level (X5), *n* (%)				
Beyond junior middle school	50 (23.70)		16 (15.38)	
High school	43 (20.38)		21 (20.19)	
Bachelor or college	44 (20.85)	25 (24.04)		
Master degree	66 (31.28)		37 (35.58)	
PhD	8 (3.79)		5 (4.81)	
Medical practitioners in family (X6), *n* (%)				
No less than one	11 (5.21)		7 (6.73)	
None	200 (94.79)		97 (93.27)	

**Table 3:** Spearman's correlations among variables.

	X1	X2	X3	X4	X5	X6	X7	X8	X9	X10	X11	X12	X13	X14	X15	X16	X17	Z1	Y1	Y2
*Medical students*																				
X1	—																			
X2	−0.01	—																		
X3	−0.14^*∗*^	0.03	—																	
X4	0.10	−0.01	−0.21^*∗∗*^	—																
X5	0.10	0.03	−0.23^*∗∗*^	0.87^*∗∗*^	—															
X6	0.00	0.03	0.03	0.23^*∗∗*^	0.29^*∗∗*^	—														
X7	0.07	−0.06	0.02	−0.07	−0.12	−0.04	—													
X8	0.12	−0.05	0.00	−0.07	−0.08	0.00	0.48^*∗∗*^	—												
X9	0.11	−0.05	0.01	−0.05	−0.10	−0.04	0.96	0.56^*∗∗*^	—											
X10	0.09	−0.06	0.01	−0.01	−0.01	−0.08	0.33^*∗∗*^	0.73^*∗∗*^	0.38^*∗∗*^	—										
X11	0.02	−0.09	−0.04	0.09	0.08	−0.04	0.17^*∗*^	0.39^*∗∗*^	0.20^*∗∗*^	0.83^*∗∗*^	—									
X12	0.02	−0.04	0.00	0.01	0.00	−0.02	0.23^*∗∗*^	0.46^*∗∗*^	0.27^*∗∗*^	0.78^*∗∗*^	0.87^*∗∗*^	—								
X13	0.09	0.00	−0.10	−0.09	−0.06	−0.05	0.19^*∗∗*^	0.12	0.19^*∗∗*^	−0.01	−0.05	−0.06	—							
X14	−0.10	−0.13	−0.01	0.08	0.07	−0.05	0.25^*∗∗*^	0.15^*∗*^	0.21^*∗∗*^	0.12	0.12	0.12	0.19^*∗∗*^	—						
X15	−0.12	−0.04	0.04	0.10	0.11	−0.01	0.15^*∗*^	0.11	0.11	0.10	0.11	0.11	0.09	0.82^*∗∗*^	—					
X16	−0.21^*∗∗*^	−0.13	0.03	−0.20^*∗∗*^	−0.21^*∗∗*^	0.03	0.42^*∗∗*^	0.12	0.43^*∗∗*^	−0.04	−0.10	−0.07	0.18^*∗∗*^	0.21^*∗∗*^	0.09	—				
X17	−0.04	−0.12	0.00	−0.13	−0.14^*∗*^	0.00	0.41^*∗∗*^	0.16^*∗*^	0.44^*∗∗*^	−0.01	−0.07	−0.04	0.30^*∗∗*^	0.26^*∗∗*^	0.16^*∗*^	0.87^*∗∗*^	—			
Z1	0.07	0.04	−0.02	−0.08	−0.07	0.07	0.26^*∗∗*^	0.20^*∗∗*^	0.26^*∗∗*^	0.10	0.08	0.10	0.12	0.19^*∗∗*^	0.14^*∗*^	0.38^*∗∗*^	0.48^*∗∗*^	—		
Y1	0.13	−0.02	−0.02	0.10	0.12	0.11	0.19^*∗∗*^	0.19^*∗∗*^	0.21^*∗∗*^	0.13	0.10	0.06	0.29^*∗∗*^	−0.10	−0.06	0.00	0.12	0.31^*∗∗*^	—	
Y2	0.26^*∗∗*^	−0.12	−0.12	0.07	0.03	−0.06	0.30^*∗∗*^	0.09	0.31^*∗∗*^	0.05	0.03	0.01	0.23^*∗∗*^	0.10	0.03	0.38^*∗∗*^	0.49^*∗∗*^	0.34^*∗∗*^	0.42^*∗∗*^	—

*Dental students*																				
X1	—																			
X2	−0.12	—																		
X3	−0.07	0.23^*∗*^	—																	
X4	0.07	0.05	0.04	—																
X5	0.11	0.00	−0.04	0.78^*∗∗*^	—															
X6	−0.16	0.04	−0.15	0.14	0.31^*∗∗*^	—														
X7	0.45^*∗∗*^	−0.09	−0.02	0.01	0.10	−0.06	—													
X8	0.44^*∗∗*^	−0.22^*∗*^	−0.01	−0.05	0.09	0.07	0.40^*∗∗*^	—												
X9	0.41^*∗∗*^	−0.07	−0.07	0.02	0.1	−0.07	0.96^*∗∗*^	0.44^*∗∗*^	—											
X10	0.22^*∗*^	−0.16	−0.01	0.09	0.19	0.12	0.24^*∗*^	0.66^*∗∗*^	0.28^*∗∗*^	—										
X11	−0.02	−0.11	−0.01	0.00	0.12	0.15	0.01	0.35^*∗∗*^	0.03	0.85^*∗∗*^	—									
X12	0.08	−0.09	−0.10	−0.02	0.07	0.07	0.16	0.47^*∗∗*^	0.20^*∗*^	0.77^*∗∗*^	0.80^*∗∗*^	—								
X13	−0.52^*∗∗*^	0.08	0.05	−0.09	−0.16	0.07	−0.14	−0.31^*∗∗*^	−0.11	−0.20^*∗*^	−0.12	−0.15	—							
X14	−0.43^*∗∗*^	0.08	−0.04	−0.09	−0.03	0.12	−0.10	−0.08	−0.08	0.04	0.04	0.02	0.40^*∗∗*^	—						
X15	−0.40^*∗∗*^	0.09	0.04	−0.11	−0.06	0.10	−0.12	−0.10	−0.12	0.06	0.09	0.06	0.40^*∗∗*^	0.91^*∗∗*^	—					
X16	0.03	−0.05	−0.16	−0.11	−0.14	0.00	0.44^*∗∗*^	0.08	0.43^*∗∗*^	0.01	−0.09	0.01	0.29^*∗∗*^	0.09	0.11	—				
X17	0.25^*∗*^	0.01	−0.13	0.00	−0.04	−0.01	0.52^*∗∗*^	0.15	0.49^*∗∗*^	0.10	−0.05	0.04	0.16	−0.06	−0.04	0.86^*∗∗*^	—			
Z1	0.15	−0.07	−0.16	0.02	−0.04	−0.03	0.41^*∗∗*^	0.13	0.37^*∗∗*^	0.16	−0.02	0.05	0.08	0.12	0.14	0.31^*∗∗*^	0.42^*∗∗*^	—		
Y1	0.30^*∗∗*^	0.03	0.02	0.14	0.14	0.05	0.62^*∗∗*^	0.31^*∗∗*^	0.59^*∗∗*^	0.16	−0.04	0.11	−0.07	−0.07	−0.12	0.31^*∗∗*^	0.47^*∗∗*^	0.44^*∗∗*^	—	
Y2	0.36^*∗∗*^	−0.01	−0.07	0.11	0.11	0.09	0.55^*∗∗*^	0.37^*∗∗*^	0.52^*∗∗*^	0.27^*∗∗*^	0.08	0.17	−0.30^*∗∗*^	−0.03	−0.06	0.20^*∗*^	0.36^*∗∗*^	0.47^*∗∗*^	0.76^*∗∗*^	—

^*∗∗*^ *p* < 0.05. ^*∗*^*p* < 0.01.

**Table 4:** Summary of multiple linear regression models.

	Medical_Y1	Dental_Y1	Medical_Y2	Dental_Y2	Medical_Z1	Dental_Z1
X1	−0.91	−3.68^*∗∗*^	1.99^*∗∗*^	1.41^*∗∗*^	−1.53	1.26
X2	2.01	0.69	0.28	0.30	−1.29	−0.15
X3	−2.69	−4.79	0.64	−1.73	1.59	−2.53
X4	−0.27	0.06	1.20	4.21	−2.11	−0.18
X5	4.41	3.92	2.34	−3.80	−0.89	−1.12
X6	2.35	−2.09	0.87	5.44	2.54	0.81
X7	13.00^*∗∗*^	0.83^*∗∗*^	−4.50^*∗∗*^	5.49^*∗∗*^	−2.89^*∗∗*^	−5.45^*∗∗*^
X8	−2.71^*∗∗*^	−8.18	5.95^*∗∗*^	6.99^*∗∗*^	13.42^*∗∗*^	−3.13
X9	−1.56^*∗∗*^	2.41^*∗∗*^	21.42^*∗∗*^	8.92^*∗∗*^	−2.09	14.76^*∗∗*^
X10	5.50	1.28	−3.43	−3.42^*∗∗*^	−0.60	3.28
X11	−1.13	−1.32	−0.75	5.35	2.90	−2.35
X12	−0.20	−3.48	−3.68	−11.28	7.73	−8.95
X13	12.89^*∗∗*^	7.31^*∗∗*^	1.41	−10.01^*∗∗*^	−3.23	3.93
X14	−19.82	−10.04	7.78	12.87	−2.34^*∗∗*^	12.14
X15	15.23	4.41	−3.70	−9.93	8.12^*∗*^	−5.76
X16	−7.63	7.88^*∗∗*^	−23.43^*∗∗*^	−10.88^*∗*^	−4.61^*∗∗*^	−15.57^*∗∗*^
X17	3.74	1.97^*∗∗*^	31.50^*∗∗*^	13.46^*∗∗*^	20.94^*∗∗*^	25.99^*∗∗*^
Z1	0.42^*∗∗*^	0.33^*∗∗*^	0.44^*∗∗*^	0.40^*∗∗*^	—	—
*R* ^*2*^	0.24	0.40	0.58	0.43	0.30	0.35
RMSE	72.36	36.98	51.13	59.95	50.81	42.16

^*∗*^ *p* < 0.05. ^*∗∗*^*p* < 0.01.

## Data Availability

The data used to support the findings of this study are restricted by the ethics review board of Tongji University in order to protect students' privacy. Data are available for researchers who meet the criteria for access to confidential data.

## References

[1] Chu C.-H., Chen F.-T., Pontifex M. B., Sun Y., Chang Y.-K. (2019). Health-related physical fitness, academic achievement, and neuroelectric measures in children and adolescents. *International Journal of Sport and Exercise Psychology*.

[2] Reed J. A., Maslow A. L., Long S., Hughey M. (2013). Examining the impact of 45 minutes of daily physical education on cognitive ability, fitness performance, and body composition of African American Youth. *Journal of Physical Activity and Health*.

[3] Ishihara T., Morita N., Nakajima T., Okita K., Sagawa M., Yamatsu K. (2018). Modeling relationships of achievement motivation and physical fitness with academic performance in Japanese schoolchildren: moderation by gender. *Physiology & Behavior*.

[4] Kinnunen M. I., Suihko J., Hankonen N., Absetz P., Jallinoja P. (2012). Self-control is associated with physical activity and fitness among young males. *Behavioral Medicine*.

[5] Syväoja H. J., Kankaanpää A., Kallio J. (2018). The relation of physical activity, sedentary behaviors, and academic achievement is mediated by fitness and bedtime. *Journal of Physical Activity and Health*.

[6] Kyan A., Takakura M., Miyagi M. (2018). Does physical fitness affect academic achievement among Japanese adolescents? A hybrid approach for decomposing within-person and between-persons effects. *International Journal of Environmental Research and Public Health*.

[7] Gil-Espinosa F. J., Cadenas-Sanchez C., Chillón P. (2019). Physical fitness predicts the academic achievement over one-school year follow-up period in adolescents. *Journal of Sports Sciences*.

[8] Pindus D. M., Drollette E. S., Scudder M. R. (2016). Moderate-to-vigorous physical activity, indices of cognitive control, and academic achievement in preadolescents. *The Journal of Pediatrics*.

[9] De Greeff J. W., Bosker R. J., Oosterlaan J., Visscher C., Hartman E. (2018). Effects of physical activity on executive functions, attention and academic performance in preadolescent children: a meta-analysis. *Journal of Science and Medicine in Sport*.

[10] Suchert V., Hanewinkel R., Isensee B. (2016). Longitudinal relationships of fitness, physical activity, and weight status with academic achievement in adolescents. *Journal of School Health*.

[11] Kahan D., Mckenzie T. L. (2017). School and neighborhood predictors of physical fitness in elementary school students. *Journal of School Health*.

[12] Tomkinson G. R., Carver K. D., Atkinson F. (2018). European normative values for physical fitness in children and adolescents aged 9-17 years: results from 2 779 165 eurofit performances representing 30 countries. *British Journal of Sports Medicine*.

[13] Estrada-Reyes C., Tlatempa-Sotelo P., Valdés-Ramos R., Cabañas-Armesilla M., Manjarrez-Montes-de-Oca R. (2018). Dietary patterns and fitness level in mexican teenagers. *Journal of Nutrition and Metabolism*.

[14] Welk G. J., Maduro S. M., Laurson K. R., Brown D. D. (2011). Field evaluation of the new FITNESSGRAM® criterion-referenced standards. *American Journal of Preventive Medicine*.

[15] Yun-Xiao L. I., Yun-Tao T. (2011). Analysis on students’ physical fitness testing result of 2011 session in Nanjing university of science and technology. *Bulletin of Sport Science & Technology*.

[16] Eliasson A. H., Lettieri C. J., Eliasson A. H. (2010). Early to bed, early to rise! sleep habits and academic performance in college students. *Sleep and Breathing*.

[17] Faught E. L., Qian W., Carson V. L. (2019). The longitudinal impact of diet, physical activity, sleep, and screen time on Canadian adolescents’ academic achievement: an analysis from the COMPASS study. *Preventive Medicine*.

[18] Wang H., Kong M., Shan W., Vong S. K. (2010). The effects of doing part-time jobs on college student academic performance and social life in a Chinese society. *Journal of Education and Work*.

[19] Cole T. J., Flegal K. M., Nicholls D., Jackson A. A. (2007). Body mass index cut offs to define thinness in children and adolescents: international survey. *BMJ*.

[20] World Health Organization (2019). *WHO Mean Body Mass Index (BMI)*.

[21] National Student Physical Health Standard, http://www.csh.edu.cn/

[22] Rampersaud G. C., Pereira M. A., Girard B. L., Adams J., Metzl J. D. (2005). Breakfast habits, nutritional status, body weight, and academic performance in children and adolescents. *Journal of the American Dietetic Association*.

[23] Adolphus K., Lawton C. L., Dye L. (2013). The effects of breakfast on behavior and academic performance in children and adolescents. *Frontiers in Human Neuroscience*.

[24] Edefonti V., Rosato V., Parpinel M. (2014). The effect of breakfast composition and energy contribution on cognitive and academic performance: a systematic review. *The American Journal of Clinical Nutrition*.

[25] Rehman R., Zafar A., Mohib A., Hussain M., Ali R. (2018). Self-reported academic performance in relation to health behaviours among Bahria University students. *The Journal of the Pakistan Medical Association*.

[26] Fekete V. K., Head M. K. (1997). Effect of breakfast consumption on academic performance of college students. *Journal of the American Dietetic Association*.

[27] Wong S. H. R., Webb T. D. (2011). Uncovering meaningful correlation between student academic performance and library material usage. *College & Research Libraries*.

[28] Alharbi A., Middleton M. (2011). The relationship between academic library usage and educational performance in Kuwait. *Library Management*.

[29] Jan S. U., Anwar M. A., Warraich N. F. (2016). Library anxiety, library use and academic performance of undergraduate students in Pakistan. *Library Review*.

[30] Brazier H., Conroy R. M. (1996). Library use and academic achievement among medical students. *Medical Education*.

[31] Andersen M. P., Mortensen R. N., Vardinghus-Nielsen H., Franch J., Torp-Pedersen C., Bøggild H. (2016). Association between physical fitness and academic achievement in a cohort of Danish school pupils. *Journal of School Health*.

[32] Cosgrove J. M., Chen Y. T., Castelli D. M. (2018). Physical fitness, grit, school attendance, and academic performance among adolescents. *BioMed Research International*.

[33] Marques A., Santos D. A., Hillman C. H., Sardinha L. B. (2018). How does academic achievement relate to cardiorespiratory fitness, self-reported physical activity and objectively reported physical activity: a systematic review in children and adolescents aged 6–18 years. *British Journal of Sports Medicine*.

[34] Chomitz V. R., Slining M. M., McGowan R. J., Mitchell S. E., Dawson G. F., Hacker K. A. (2010). Is there a relationship between physical fitness and academic achievement? Positive results from public school children in the northeastern United States. *Journal of School Health*.

[35] Lipošek S., Planinšec J., Leskošek B., Pajtler A. (2019). Physical activity of university students and its relation to physical fitness and academic success. *Annales Kinesiologiae*.

[36] Qi F., Qing-Le Z., Yue D., Yong-Ling Y., Qi-Qiang H., Robert S. (2014). Associations of physical activity, screen time with depression, anxiety and sleep quality among Chinese college freshmen. *PLoS One*.

[37] Al-Drees A., Abdulghani H., Irshad M. (2016). Physical activity and academic achievement among the medical students: a cross-sectional study. *Medical Teacher*.

